# Maternal Cadmium Levels during Pregnancy Associated with Lower Birth Weight in Infants in a North Carolina Cohort

**DOI:** 10.1371/journal.pone.0109661

**Published:** 2014-10-06

**Authors:** Jill E. Johnston, Ellis Valentiner, Pamela Maxson, Marie Lynn Miranda, Rebecca C. Fry

**Affiliations:** 1 Department of Environmental Sciences and Engineering, Gillings School of Global Public Health, University of North Carolina at Chapel Hill, Chapel Hill, North Carolina, United States of America; 2 Department of Epidemiology, Gillings School of Global Public Health, University of North Carolina at Chapel Hill, Chapel Hill, North Carolina, United States of America; 3 School of Natural Resources and Environment, University of Michigan, Ann Arbor, Michigan, United States of America; 4 Department of Pediatrics, University of Michigan, Ann Arbor, Michigan, United States of America; 5 Department of Obstetrics and Gynecology, University of Michigan, Ann Arbor, Michigan, United States of America; University of Cincinnati, United States of America

## Abstract

Cadmium (Cd) is a ubiquitous environmental contaminant, a known carcinogen, and understudied as a developmental toxicant. In the present study, we examined the relationships between Cd levels during pregnancy and infant birth outcomes in a prospective pregnancy cohort in Durham, North Carolina. The study participants (n = 1027) had a mean Cd level of 0.46 µg/L with a range of <0.08 to 2.52 µg/L. Multivariable models were used to establish relationships between blood Cd tertiles and fetal growth parameters, namely birth weight, low birth weight, birth weight percentile by gestational age, small for gestational age, pre-term birth, length, and head circumference. In multivariable models, high maternal blood Cd levels (≥0.50 µg/L) during pregnancy were inversely associated with birth weight percentile by gestational age (p = 0.007) and associated with increased odds of infants being born small for gestational age (p<0.001). These observed effects were independent of cotinine-defined smoking status. The results from this study provide further evidence of health risks associated with early life exposure to Cd among a large pregnancy cohort.

## Introduction

Cadmium (Cd) is a heavy metal ubiquitous in the environment and understudied as a developmental toxicant. In animal models, Cd is embryotoxic and teratogenic [1]. In humans, Cd is a known lung carcinogen and exposure has been linked to adverse effects on the liver, kidney, bladder, stomach and pancreas [2], [3], [4]. Furthermore, there is growing evidence of associations between Cd and adverse birth outcomes, such as small for gestational age and preterm birth [5], [6], [7], [8], [9], [10]. In addition to its impact on growth parameters, Cd is an established epigenetic modifier [11], thus there is the potential that prenatal exposure to Cd may also have long-term implications on child development.

The largest source of Cd exposure is through tobacco products. An average cigarette contains 1–2 µg of Cd, of which smokers absorb a portion via inhalation [12]. One cigarette has been shown to increase the blood Cd level by approximately 0.1–0.2 µg/L [13]. In the context of smoking, cotinine, the primary metabolite of nicotine and a biomarker of cigarette smoke exposure, has a half-life of less than one day [14], [15]. Thus, cotinine is a general measure of recent cigarette exposure. While tobacco products are the largest source of exposure, diet is the most common exposure source. Cd can be found at elevated levels in food such as shellfish, organ meats, cereals, root vegetables, and green leafy vegetables [16], [17]. It is estimated that the average Cd intake from food varies between 8 and 25 µg per day [4]. Cd exposure may also occur via house dust and consumer products, as well as due to proximity to industrial sources [4], [18], [19], [20]. The half-life of Cd in blood can range up to 10 years and thus may also serve as a good reflection of historical exposures [4], [13].

Limited information is available on Cd levels among pregnant women in the United States. A recent study among approximately 200 pregnant women in central North Carolina found Cd blood levels ranging up to 2.79 µg/L with a geometric mean of 0.18 µg/L [21]. Furthermore, average Cd blood levels among a pregnant women subsample of the 2003–2004 National Health and Nutrition Examination Survey (NHANES) were similar with a mean of 0.22 µg/L (n = 253) compared to 0.33 µg/L (n = 1396) among non-pregnant women of childbearing age [22].

In this paper, we assess the relationship between Cd levels among pregnant women and birth outcomes. While smoking is well-established as a cause of adverse birth outcomes as well as a source of Cd in blood, we hypothesized that Cd has detrimental effects on fetal health independent of smoking. As both Cd and cotinine were assessed, we were able to examine the relationship of Cd independently on measured birth outcomes.

## Materials and Methods

### Study population

The Healthy Pregnancy, Healthy Baby Study is a prospective cohort study that enrolled pregnant women living in Durham County, North Carolina, U.S., from 2005 to 2010. The study, part of the Southern Center on Environmentally Driven Disparities in Birth Outcomes (SCEDDBO), aims to examine the effects of environmental, social, and host factors on racial disparities in pregnancy outcomes. Women receiving prenatal care at either the Duke University Obstetrics Clinic or the Durham County Public Health's Prenatal Clinic were eligible to participate if they were between 18 and 28 weeks of gestation, were at least 18 years of age, were English-literate, lived in Durham County, did not have a multi-fetal gestation or any known congenital anomalies, and planned to deliver at Duke University Medical Center (DUMC). Of the 2,306 women approached to participate in the study, 1,897 (82.3%) women enrolled. Women were excluded if they were less than 18 years old, non-English literate, lived outside of Durham County, were less than 18 or greater than 28 weeks of gestation at the time of enrollment, had a multi-fetal gestation, had a known fetal genetic or congenital anomaly, or were not planning to deliver at DUMC. Study participants gave written documentation of consent for participation. This study was reviewed and approved by the Duke University Institutional Review Board (Pro00007633).

Previously, this cohort was used to examine lead and mercury exposures among pregnant women [23], [24]. Because the subset of women for whom lead, mercury, and Cd exposure measurements are available varies, we cannot directly connect Cd exposure levels from this study to the exact same populations in the previous studies.

### Blood Cd and cotinine levels

Maternal blood samples were collected at the time of delivery in a Monoject trace element blood collection tube containing the anticoagulant EDTA. Whole blood samples were analyzed by Inductively Coupled Plasma-Mass Spectrometry at the Mayo Clinical Laboratories (MCL) or the Dwyer (Duke) labs for Cd. Plasma samples were analyzed at the MCL or University of California, San Francisco (UCSF) Clinical Pharmacology Laboratory for cotinine. The limits of detection (LOD) for Cd were 0.2 and 0.08 µg/L at the MCL and Duke Laboratories, respectively, with 29.9% below the LOD at the MCL and 3.5% below the LOD at Duke Laboratories. The LOD for cotinine at the MCL was 2.0 µg/L with 75.4% below the LOD and was 0.02 ug/L with 16.4% below the LOD at UCSF. Because women vary in timing of delivery, blood samples were collected from 23–42 weeks of gestation, with a median gestational age of 39 weeks (interquartile range: 37–39 weeks).

Since the labs that analyzed the samples for both Cd and cotinine levels changed over the course of the study, the results were normalized across labs and values below the detection limits were imputed using the rank permutation method proposed by Burgette and Reiter [25]. Blood cotinine levels above 10 µg/L are indicative of active smoking [26], [27]. This cutoff was used to define participants as smokers or non-smokers. For the purposes of this study, blood Cd levels were classified as low (≤0.28 µg/L), medium (0.29–0.49 µg/L) or high (≥0.50 µg/l) based on tertiles.

### Birth outcomes

Birth outcome information, including birth weight, newborn length, gestational age and head circumference, were obtained through the infant's electronic medical record. Births were dichotomized into low birth weight (LBW; <2500 g) and pre-term birth (PTB; <37 weeks gestational age). Birth weight percentile was calculated based upon national normalized birth weight for gestational age and sex, and dichotomized based on small for gestational age (SGA) defined at <10^th^ percentile. Both continuous and binary outcome measures were used for analysis because most increases in birth weight are associated with better infant health and development outcomes [28], [29].

### Sociodemographic and patient characteristics

Demographic, health behavior, and medical history data were obtained by direct patient interview and through electronic medical record review at the time of enrollment. Age was trichotomized as <20 years, 20–34 years, and ≥35 years. Educational attainment was classified as no high school degree, completed high school, and schooling beyond high school. We dichotomized patient insurance status as having private insurance or not having private insurance (i.e., participants who were either uninsured or covered by Medicaid or other public health insurance). Lack of private insurance was considered a proxy for lower socioeconomic status (SES) [30].

### Data restrictions

The full study cohort (n = 1,852) was restricted to non-Hispanic black and non-Hispanic white participants with an imputed blood Cd level per the transformation described above, leaving 1438 eligible women. An additional 408 participants were excluded due to missing blood cotinine levels, and three participants with missing covariate data were excluded. After these exclusions the study sample size was n = 1027. The sample size varies in some analyses due to missingness in birth outcome data.

### Statistical analysis

Exploratory analyses were conducted to describe blood Cd levels by patient characteristics. Cohen's κ was used to assess agreement between reported and blood-cotinine-defined smoking status. Unadjusted relationships between birth outcomes and patient characteristics were explored using chi-square and nonparametric one-way tests. For binary outcomes (e.g. SGA) we characterized associations using chi-square tests and odds ratios, and for continuous outcomes (e.g. birth weight), we used two-sample t-tests and analysis of variance (ANOVA) with Tukey-Kramer corrections for multiple comparisons. A Satterthwaite approximation was used for the degrees of freedom for comparisons in which group variances were heterogeneous; with a test statistic denoted as *t_s_*_._

In order to assess the relationship between Cd and birth outcomes, linear regression models were fit for continuous measures of birth outcomes and logistic regression models for dichotomous outcomes, controlling for race, age, education, insurance status, parity, history of anxiety, cotinine defined smoking status, and infant sex. Since blood cotinine levels may covary with Cd, models were fit with and without an interaction term between the two exposures. All analyses were conducted using SAS 9.3 (SAS Institute, Cary, NC).

## Results

### Characteristics of the study population

The demographic composition of the study population is presented in **Table 1**. Of the 1,027 participants meeting all restrictions, the majority was non-Hispanic Black (74.2%), had previously given birth (74.9%) and lacked private insurance (71.1%). Roughly 87% had a minimum of a high school education. On average, the participants were 26 years old, ranging from 18–40 years old. Nine percent of the participants had a history of anxiety.

**Table 1:** Study population characteristics.

	N (Column)				
	All (Column %)	Low Blood Cd (n = 343)	Medium Blood Cd (n = 354)	High Blood Cd (n = 330)	p*
	(n = 1027)				
**Age (years)**					0.1242
<20	137 (13)	54 (16)	50 (14)	33 (10)	
20–34	748 (73)	250 (73)	254 (72)	244 (74)	
≥35	142 (14)	39 (11)	50 (14)	53 (16)	
**Race**					0.0518
non-Hispanic white	266 (26)	103 (30)	91 (26)	72 (22)	
non-Hispanic black	761 (74)	240 (70)	263 (74)	258 (78)	
**Education Level**					<0.0001
<High school	135 (13)	35 (10)	35 (10)	65 (20)	
High school	360 (35)	113 (33)	122 (35)	125 (38)	
> High school	532 (52)	195 (57)	197 (56)	140 (42)	
**Relationship status**					0.2325
Committed rel.	760 (74)	259 (76)	268 (76)	233 (71)	
No committed rel.	267 (26)	84 (24)	86 (24)	97 (29)	
**Insurance status**					0.0003
Private insurance	290 (28)	118 (34)	104 (29)	68 (21)	
No private insurance	737 (72)	225 (66)	250 (71)	262 (79)	
**History of anxiety**					0.6558
No	928 (90)	308 (90)	324 (92)	29 (90)	
Yes	99 (10)	35 (10)	30 (8)	34 (10)	
**Parity status**					0.0067
Pluriparous	771 (75)	237 (69)	274 (77)	260 (79)	
Nulliparous	256 (25)	106 (31)	80 (23)	70 (21)	
**Infant**'**s sex**					0.1417
Male	512 (50)	167 (49)	191 (54)	154 (47)	
Female	515 (50)	176 (51)	163 (46)	176 (53)	
**Blood cotinine**					<0.0001
≤ 10 µg/L	771 (75)	312 (91)	287 (81)	172 (52)	
> 10 µg/L	256 (25)	31 (9)	67 (19)	156 (48)	

Note: Percentages are reported as column-wise. Blood cadmium levels: low – ≤0.28 µg/L, medium – 0.29–0.49 µg/L, high – ≥0.50 µg/l.

* p-values represent results from chi-square tests.

Self-reported smoking combined with medical record data identified 169 (16.4%) participants as current or past smokers. Based on blood cotinine levels exceeding 10 µg/L, 24.8% of participants were classified as active smokers during pregnancy. Agreement between the two measures of smoking status was substantial (κ = 0.639, *p*<.0001), and all subsequent analyses presented here use cotinine-defined smoking status.

### Cd levels in the study population

The mean blood Cd level was 0.46 µg/L (SD 0.34 µg/L), and the 50^th^ percentile was 0.40 µg/L with an inter-quartile range of 0.33 µg/L. Over 60% of the study population had a blood Cd level above the mean for US adults (0.32 µg/L), and 77.7% had a blood Cd level greater than the mean for pregnant women (0.22 µg/L) [31]. This indicates that this Durham County study population had higher levels of Cd exposure than expected given national data. Since blood Cd levels in our study population do not reflect national levels, blood Cd levels were classified based on tertiles. In all subsequent analyses blood Cd levels are referred to as low (≤0.28 µg/L), medium (0.29–0.49 µg/L), or high (≥0.50 µg/l).

Chi-square tests showed differences in blood Cd levels based on education, insurance status, parity, and cotinine-defined smoking status (see **Table 1**). No differences were observed by age, race, relationship status, history of anxiety, parity, or infant sex.

Compared to non-smokers, smokers had 2.34 (95% CI: 1.50, 3.74) increased odds of having a medium blood Cd level and 3.87 (95% CI: 2.79, 5.49) increased odds of having a high blood Cd level compared to a low blood Cd level. Smokers (versus nonsmokers) had a 9.06 (95% CI: 5.98, 14.13) increased odds of having a high versus medium blood Cd level.

### Unadjusted association between birth outcomes and Cd

Birth outcomes are summarized by study population demographics and blood Cd levels in **Table 2**. The average weight of the newborns was 3095 g (SD 651 g) and ranged from 500 g to 5160 g. Birth weight was lower among infants whose mothers were non-Hispanic black (*p*<.0001), without private insurance (*p*<.0001), with elevated blood cotinine levels (*p* = 0.0007), and with high blood Cd levels (*p* = 0.0203; High – Low, *p* = 0.0148). Additionally, male infants weighed more than females (*p* = 0.0012), which is consistent with national data.

**Table 2:** Cross-tabulation of birth outcomes and maternal blood cadmium and cotinine levels.

	N (Column %)	Mean (SD)					N (Row %)		
	All	Birth weight (g)	Gest. age (weeks)	Birth weight percentile	Head circ. (cm)	Length (cm)	LBW (<2500 g)	SGA	PTB (<37 weeks)
All	1027	3095.2	38.1 (2.5)	39.3	33.4	48.3	148	154	153
	−100	−651		−27.59	−2.5	−3.6	−14	−15	−15
**Age (years)**									
<20	137	3045.4	38.4 (2.4)	35.2	33.5	48.3	28	28	19
	−13	−616.1		−25.9	−2.8	−3.2	−20	−20	−14
20−34	748	3093	38.1 (2.5)	39	33.3	48.2	101	114	104
	−73	−652.1		−27.8	−2.4	−3.7	−14	−15	−14
≥35	142	3154.4	37.9 (2.6)	44.6	33.8	48.4	19	12	30
	−14	−677.9		−27.4	−2.7	−3.5	−13	−9	−21
**Race**									
NHW	266	3325.8	38.4 (2.1)	49.9	34.3	49.4	17	12	30
	−26	−572.2		−27.5	−2.1	−2.8	−6	−5	−11
NHB	761	3014.6	38.0 (2.7)	35.6	33.1	47.9	131	142	123
	−74	−657.9		−26.7	−2.6	−3.7	−17	−19	−16
**Education**									
<HS	135	3100	38.1 (2.3)	38.6	33.4	48.1	23	26	18
	−13	−670.9		−29	−2.7	−3.5	−17	−19	−13
HS	360	3043.2	38.2 (2.5)	35.5	33.2	48	55	60	48
	−35	−619		−26	−2.3	−3.7	−15	−17	−13
>HS	532	3129.1	38.0 (2.6)	42	33.5	48.5	70	68	87
	−52	−665.8		−28	−2.6	−3.5	−13	−13	−16
**Relationship status**									
Committed rel.	760	3113.8	38.1 (2.6)	40.5	33.5	48.3	102	99	115
	−74	−651.4		−27.5	−2.6	−3.7	−13	−13	−15
No committed rel.	267	3042.1	38.2 (2.4)	36	33.3	48.1	46	55	38
	−26	−648.3		−27.7	−2.4	−3.1	−17	−21	−14
**Insurance status**									
Private insurance	290	3253.9	38.2 (2.3)	48.3	34	49	25	22	42
	−28	−607.6		−27.6	−2.3	−3.1	−9	−8	−14
No private insurance	737	3032.7	38.1 (2.6)	35.8	33.2	47.9	123	132	111
	−72	−657.3		−26.8	−2.6	−3.7	−17	−18	−15
**History of Anxiety**									
No	928	3105.6	38.1 (2.5)	39.7	33.4	48.3	131	137	135
	−90	−649.3		−27.7	−2.6	−3.6	−14	−15	−15
Yes	99	2997.7	37.7 (2.8)	36	33.3	47.9	17	17	18
	−10	−662.4		−26.7	−2.2	−3.4	−17	−17	−18
**Parity**									
Nulliparous	771	3100.1	38.0 (2.6)	40.2	33.4	48.2	112	110	121
	−75	−671.2		−28.2	−2.5	−3.4	−15	−14	−16
Pluriparous	256	3080.2	38.4 (2.3)	36.6	33.3	48.4	36	44	32
	−25	−587		−25.6	−2.7	−4	−14	−17	−13
**Infant**'**s sex**									
Male	512	3160.9	38.0 (2.6)	40.8	33.6	48.5	64	61	82
	−50	−664		−27	−2.7	−3.9	−13	−12	−16
Female	515	3029.9	38.2 (2.4)	37.8	33.2	48	84	93	71
	−50	−631.8		−28.1	−2.3	−3.2	−16	−18	−14
**Cotinine> 10 ng/ml**									
No	771	3134.9	38.1 (2.5)	41.6	33.5	48.5	98	98	117
	−75	−645		−27.7	−2.6	−3.6	−13	−13	−15
Yes	256	2975.6	38.0 (2.6)	32.3	33.1	47.6	50	56	36
	−25	−655.8		−26.2	−2.4	−3.4	−20	−22	−14
**Blood Cd**									
Low	343	3166.3	38.2 (2.3)	42.8	33.5	48.6	43	41	45
	−33	−639.2		−28.3	−2.2	−3.5	−13	−12	−13
Medium	354	3090.2	37.9 (2.9)	40.9	33.5	48.1	52	43	58
	−35	−697.3		−27.5	−2.7	−4	−15	−12	−16
High	330	3026.6	38.2 (2.3)	33.9	33.2	48.1	53	70	50
	−32	−604.4		−26.2	−2.6	−3.2	−16	−21	−15

Note: The percentages in the total column are reported column-wise, all other percentages in this table are row-wise. Blood cadmium levels: low – ≤0.28 µg/L, medium – 0.29–0.49 µg/L, high – ≥0.50 µg/l.

Of the infants, 148 (14.41%) were considered low birth weight (LBW). Women under 20 years had 1.65 (95% CI: 1.03, 2.62) times the odds of having a LBW child compared to women 20–34 years, but there was no statistical difference between women <20 and ≥35 years or 20–34 and ≥35 years. LBW was 3.05 (95% CI: 1.80, 5.15) times more frequent among non-Hispanic blacks than non-Hispanic whites. Women without private insurance were 2.12 (95% CI: 1.35, 3.34) times as likely to have LBW as women with private insurance. Elevated levels of cotinine during pregnancy corresponded to 1.67 (95% CI: 1.15, 2.42) times the odds of a LBW infant compared to blood cotinine levels below 10 µg/l. There was no relationship between increased odds of LBW and Cd levels.

Gestational age at delivery ranged from 23 weeks to 42 weeks with a mean of 38.1 weeks (SD 2.5 weeks). Gestational periods were slightly shorter among non-Hispanic blacks (*p* = 0.045) and nulliparous women (*p* = 0.0080). In unadjusted models, gestational age at delivery did not vary by blood cotinine or blood Cd levels.

Birth weight percentile for gestational age and sex ranged from the 0^th^ to 99.5^th^ percentiles with the average at the 39.30^th^ percentile (SD 27.59 percentiles). There were significant relationships between birth weight percentile and mother's age (*p* = 0.0159), race (*p*<.0001), education (*p* = 0.0025), relationship status (*p* = 0.0227), insurance status (*p*<.0001), cotinine-defined smoking status (*p*<.0001), and blood Cd levels (*p*<.0001). High blood Cd levels were associated with significantly lower age- and sex-adjusted birth weight percentiles compared to medium blood Cd levels (*p* = .0025) and low blood Cd levels (*p*<.0001). There was not a significant difference between women with medium and low blood Cd levels.

There were 154 (15%) infants born small for gestational age (SGA). Relative to women at least 35 years old, SGA births were 2.78 (95% CI: 1.35, 5.73) times more frequent among women younger than 20, and 1.95 (95% CI: 1.04, 3.64) times more frequent among women 20–34 years. Non-Hispanic black women were 4.86 (95% CI: 2.65, 8.91) times as likely as non-Hispanic white women to give birth to an SGA infant. Women without private health insurance were 2.66 (95% CI: 1.66, 4.27) more likely to have an SGA infant compared to women with private health insurance. Elevated blood cotinine levels corresponded to 1.92 (95% CI: 1.34, 2.77) times the odds of SGA. High blood Cd levels corresponded to 1.98 (95% CI: 1.30, 3.02) times the odds of SGA compared to low blood Cd levels, and 1.95 (95% CI: 1.29, 2.95) times the odds of SGA compared to medium blood Cd levels.

In addition, the study cohort includes 153 infants (15%) who were born pre-term (PTB). Relative to women 20–34 years, PTB infants were 1.66 (95% CI: 1.05, 2.61) times more frequent among women at least 35 years old. There was no difference in odds of PTB based on maternal cotinine or Cd levels.

Mean head circumference was 33.40 cm (SD 2.52 cm) ranging from 15–49.5 cm among the infants. Head circumference was lower among infants whose mothers were non-Hispanic black (*p*<.0001), without private insurance (*p*<.0001), and among cotinine-defined smokers (*p* = 0.0265). Newborn length ranged from 19.5–58.0 cm with an average of 48.27 (SD 3.57 cm). Infant newborn length was lower for women who were non-Hispanic black (*p*<.0001), without private insurance (*p*<.0001), and cotinine-defined smokers (*p* = 0.0009). Neither head circumference nor length was associated with blood Cd levels.

### Adjusted association between birth outcomes and Cd

Multivariable regression was used to further examine these relationships while adjusting for confounders including maternal age, education, and pregnancy history. Consistent with the unadjusted analysis, Cd levels were not associated with preterm birth, head circumference, or length (**Tables 3** **and** **4**). However significant associations between Cd levels and birth weight percentile by gestational age and SGA were identified (**Tables 3** **and** **4**).

**Table 3:** Adjusted estimates and standard errors for continuous birth outcomes.

	Beta (SE)				
	Birth weight	Gest. age	Birth weight percentile for gestational age (n = 1027)	Head circ.	Length
	(n = 1027)	(n = 1027)		(n = 992)	(n = 994)
**Blood Cd: High**	−79.7 (52.8)	0.09 (0.21)	−6.11 (2.21)**	−0.13 (0.21)	−0.04 (0.30)
**Blood Cd: Medium**	−63.0 (48.3)	−0.29 (0.19)	−1.23 (2.02)	−0.02 (0.19)	−0.33 (0.27)
**Blood cotinine> 10 µg/L**	−98.1 (52.3)	−0.08 (0.21)	−5.18 (2.19)*	−0.18 (0.21)	−0.63 (0.30)*
**Age <20 years**	−0.08 (62.8)	0.27 (0.25)	−0.75 (2.63)	0.38 (0.25)	0.31 (0.35)
**Age ≥34 years**	−46.0 (61.9)	−0.29 (0.25)	0.01 (2.59)	0.02 (0.25)	−0.45 (0.35)
**Race: NHB**	−293.2 (54.7)***	−0.67 (00.22)**	−11.30 (2.29)***	−1.07 (0.22)***	−1.51 (0.31)***
**Education: <HS**	77.8 (65.0)	−0.08 (0.26)	4.43 (2.72)	0.13 (0.26)	0.13 (0.36)
**Education:> HS**	−30.4 (48.5)	−0.18 (0.19)	0.36 (2.03)	−0.08 (0.19)	−0.08 (0.27)
**No private insurance**	−81.1 (57.8)	0.05 (0.23)	−5.92 (2.42)*	−0.36 (0.23)	−0.38 (0.32)
**Parity status: Nulliparous**	63.9 (48.0)	−0.33 (0.19)	−5.72 (2.01)**	0.27 (0.19)	0.14 (0.27)
**Infant**'**s sex: Female**	−113.5 (39.5)**	0.19 (0.16)	-	−0.34 (0.16)*	−0.5 (0.22)*
**History of anxiety: Yes**	−187.8 (68.5)**	−0.48 (0.27)	−7.58 (2.87)**	−0.46 (0.27)	−0.78 (0.38)*
**Intercept**	3481.3 (78.0)***	38.9 (0.31)***	57.92 (3.04)***	34.52 (0.31)***	50.20 (0.44)***

Note: estimates are adjusted for the other model covariates; infant's sex was excluded as a covariate from the birth weight percentile for gestational age model since the birth weight percentiles are sex-adjusted. Blood cadmium levels: low – ≤0.28 µg/L, medium – 0.29–0.49 µg/L, high – ≥0.50 µg/l.

* p<0.05, ** p<0.01, ***p<0.001.

**Table 4:** Adjusted odds ratios and 95% confidence intervals for dichotomous birth outcomes.

	Odds ratio (95% confidence interval)		
	LBW	SGA	PTB
	(n = 1027)	(n = 1027)	(n = 1027)
**Blood Cd: High**	1.07 (0.67, 1.73)	1.72 (1.1, 2.68)*	1.17 (0.74, 1.87)
**Blood Cd: Medium**	1.13 (0.73, 1.76)	0.99 (0.63, 1.55)	1.24 (0.81, 1.89)
**Blood cotinine> 10 µg/L**	1.49 (0.96, 2.29)	1.41 (0.94, 2.12)	0.90 (0.57, 1.42)
**Age <20 years**	1.55 (0.93, 2.56)	1.25 (0.76, 2.04)	1.08 (0.62, 1.88)
**Age ≥34 years**	1.39 (0.79, 2.46)	0.73 (0.39, 1.39)	1.72 (1.06, 2.82)*
**Race: NHB**	2.89 (1.59, 5.26)***	4.38 (2.3, 8.35)***	1.96 (1.18, 3.28)*
**Education: <HS**	1.06 (0.61, 1.82)	1.12 (0.67, 1.86)	1.01 (0.56, 1.81)
**Education:> HS**	1.25 (0.82, 1.91)	1.20 (0.8, 1.79)	1.37 (0.9, 2.08)
**No private insurance**	1.32 (0.76, 2.30)	1.22 (0.71, 2.09)	1.03 (0.63, 1.68)
**Parity status: Nulliparous**	0.98 (0.64, 1.52)	0.72 (0.48, 1.08)	1.23 (0.80, 1.90)
**Infant**'**s sex: Female**	1.31 (0.92, 1.87)	1.50 (1.07, 2.12)*	0.85 (0.6, 1.20)
**History of anxiety: Yes**	1.56 (0.87, 2.80)	1.67 (0.94, 2.97)	1.43 (0.81, 2.50)

Note: estimates are adjusted for the other model covariates; LBW – low birth weight, SGA – small for gestational age, PTB – preterm birth; Blood cadmium levels: low – ≤0.28 µg/L, medium – 0.29–0.49 µg/L, high – ≥0.50 µg/l.

* p<0.05, ***p<0.001.

Controlling for smoking status and other demographic variables, on average, pregnant women in the high Cd exposure group were found to have infants 6.1 birth weight for gestational age percentile points lower (95% CI: −10.5 to −1.8) than the low Cd exposure group (*p* = 0.0057) and were 1.71 (95% CI: 1.10, 2.64) times as likely to be SGA. While the medium Cd exposure tertile was not statistically significantly different from the low exposure group, a negative monotonic relationship was observed between Cd exposure group and birth weight percentile. Smoking was also significantly associated with lower birth weight percentile by gestational age with a similar magnitude as high Cd exposure (−5.2; 95% CI: −9.5 to −0.9; *p* = 0.0179). Race exhibited the largest effect on birth weight percentile, with non-Hispanic black women, on average, having an infant 11.2 birth weight for gestational age percentile points lower (CI: −15.7 to −6.7) than non-Hispanic white women. Non-Hispanic black women were also 4.42 (95% CI: 2.34, 8.35) times more likely to have an SGA infant. Pregnant women without private health insurance, as well as nulliparous women, had, on average, lower birth weight infants. No differences were observed by age or educational status.

## Discussion

Low birth weight is an important public health problem globally, associated with neonatal mortality and morbidity, as well as increased disease risk later in life [32]. This study contributes to the growing evidence on the relationship between maternal exposure to Cd during pregnancy and adverse pregnancy outcomes [7], [10], [33]. Among this large urban and minority cohort of women pregnant women, over 60% have blood Cd levels that exceed the median for US adults as reported in NHANES [31]. These levels are likely a result of a combination of exposure sources, including but not limited to cigarette smoke. While 25% of the pregnant women were active smokers based on blood cotinine levels, elevated blood Cd levels were still observed in the non-smokers ranging from <0.08–2.26 µg/L. In this study, which evaluated the association between Cd and birth outcomes, we observed an inverse relationship between Cd exposure and birth weight (as a percentile and as SGA). Specifically, we show that infants born to women with blood Cd in the highest tertile of exposure (≥0.50 µg/L) were born on average 6 birth weight for gestational age percentile points lower compared to the lowest Cd exposed group. In addition, infants born to women in the highest Cd exposure tertile were 1.71 times as likely to be born small for gestational age.

There are various possible mechanisms by which Cd exposure during pregnancy can limit fetal growth. Importantly, prior investigations have demonstrated that while Cd accumulates in the human placenta, the placenta is not a complete barrier [34]. As a result, concentrations of Cd in cord blood representing fetal levels increase with maternal exposure [5], [35]. In addition, Cd levels in cord blood and in the placenta are strongly correlated with maternal blood levels [5], [36]. Infant birth weight may be negatively influenced by Cd levels as a result of the indirect toxic effects of the metal on the placenta or direct effects on the fetus. It is hypothesized that the higher Cd levels can lead to insufficient transfer of zinc to the fetus, which can retard intrauterine growth [35], [37], [38]. Another possible mechanism is through the endocrine disrupting properties of Cd which may reduce *in utero* gene and subsequently protein expression linked to fetal growth [39].

While we observe a relationship between Cd exposure independent of smoking and lower birth weight percentile and SGA among pregnant women in NC, this study is not without limitations. The study population comes from pregnant women in one urban county in North Carolina that intentionally oversampled for non-Hispanic black women and may not be representative of Cd levels among pregnant women in other areas. This study used maternal blood levels as a proxy for exposure to the fetus via cord blood or the placenta. While maternal blood Cd levels are strongly correlated with Cd in cord blood and in the placenta, direct measures of infant Cd levels would provide a more direct measure of the association between fetal Cd exposure and birth outcomes. Additionally, the laboratories used to analyze samples for both Cd and cotinine levels changed over the course of the study. Although we addressed the change in labs using a rank permutation method to transform values to the same scale, the normalized values may differ from the true scale; therefore we can only make inferences about relative Cd exposure. Data were not available on dietary information or ambient Cd concentration, two important sources of Cd exposure, particularly among the non-smoking cohort. Regardless of knowing exposure sources, this study provides evidence that maternal blood Cd is associated with adverse birth outcomes. Future research is needed to identify the direct sources of exposure among non-smokers in order to design appropriate inventions and improve public health.

## Conclusions

We observed a significant negative association between maternal Cd exposure and birth weight. This study suggests that Cd impairs fetal growth, independent of the contribution of smoking. These findings add to the growing body of literature supporting the negative effects of fetal Cd exposure on birth outcomes. Additionally, they highlight the presence of elevated levels of Cd in pregnant women in NC, and highlight the need for understanding the role of dietary, occupational, environmental, lifestyle and host factors that influence Cd exposure levels. Such information is important for the development of successful invention programs aimed at reducing exposures and narrowing disparities in birth outcomes.
